# The Cerrado (Brazil) plant cytogenetics database

**DOI:** 10.3897/CompCytogen.11(2).11395

**Published:** 2017-04-25

**Authors:** Fernando Roa, Mariana Pires de Campos Telles

**Affiliations:** 1 Programa de pós-graduação em Genética e Melhoramento de Plantas, Escola de Agronomia, Universidade Federal de Goiás, 74001-970, Goiânia, GO, Brazil; 2 Escola de Ciências Agrárias e Biológicas, Pontifícia Universidade Católica de Goiás, 74605-010, Goiânia, GO, Brazil

**Keywords:** Cerrado, cytogenetics, database, scientometrics, shiny-R

## Abstract

Cerrado is a biodiversity hotspot that has lost ca. 50% of its original vegetation cover and hosts ca. 11,000 species belonging to 1,423 genera of phanerogams. For a fraction of those species some cytogenetic characteristics like chromosome numbers and C-value were available in databases, while other valuable information such as karyotype formula and banding patterns are missing. In order to integrate and share all cytogenetic information published for Cerrado species, including frequency of cytogenetic attributes and scientometrics aspects, Cerrado plant species were searched in bibliographic sources, including the 50 richest genera (with more than 45 taxa) and 273 genera with only one species in Cerrado. Determination of frequencies and the database website (http://cyto.shinyapps.io/cerrado) were developed in R. Studies were pooled by employed technique and decade, showing a rise in non-conventional cytogenetics since 2000. However, C-value estimation, heterochromatin staining and molecular cytogenetics are still not common for any family. For the richest and best sampled families, the following modal 2n counts were observed: Oxalidaceae 2n = 12, Lythraceae 2n = 30, Sapindaceae 2n = 24, Solanaceae 2n = 24, Cyperaceae 2n = 10, Poaceae 2n = 20, Asteraceae 2n = 18 and Fabaceae 2n = 26. Chromosome number information is available for only 16.1% of species, while there are genome size data for only 1.25%, being lower than the global percentages. In general, genome sizes were small, ranging from 2C = ca. 1.5 to ca. 3.5 pg. Intra-specific 2n number variation and higher 2n counts were mainly related to polyploidy, which relates to the prevalence of even haploid numbers above the mode of 2n in most major plant clades. Several orphan genera with almost no cytogenetic studies for Cerrado were identified. This effort represents a complete diagnosis for cytogenetic attributes of plants of Cerrado.

## Introduction

Cerrado, a phytogeographic domain from Brazil, is the third largest biodiversity hotspot in the world considering species endemism and degree of threat (Myers et al. 2000; Forzza et al. 2012). In fact, it has suffered more anthropic impact than the Amazonian tropical forest (Spera et al. 2016). With originally 2m km^2^ of wilderness area, of which ca. 50% are currently cultivated, this region hosts ca. 11,000 species belonging to 1,423 genera of 171 families of phanerogams (Sano et al. 2008; Ferreira et al. 2016). Classical cytogenetic studies, comprehending somatic or meiotic chromosome counts are known for a considerable portion of species occurring in Cerrado but in many cases they were not performed on plants from this region. Meanwhile, studies of genome size, differential staining and molecular cytogenetics are very rare.

Since the chromosome number is the most basic karyotype feature and it can be observed by conventional staining of meristem cells or pollen mother cells, most cytogenetic databases deal only with this attribute (Peruzzi and Bedini 2014; Rice et al. 2015). Estimates of genome size, which are currently addressed mostly by comparing the relative fluorescence of propidium iodide stained nuclei measured in a flow cytometer to that of known patterns, are compiled in the Kew C-value database (Doležel and Greilhuber 2010; Garcia et al. 2014a). Other valuable information such as: karyotype formula, dependent on the relative position of centromere along the chromosome and chromosome length (Guerra 1986), silver staining of nucleolar organizer regions (AgNOR) (Vieira et al. 1990), and heterochromatin staining after treatment with acids, bases or denaturation (Schwarzacher et al. 1980; Guerra 2000; Barros e Silva and Guerra 2010) are not included in databases. In contrast, fluorescence in situ hybridization (FISH), in which a DNA probe produced by molecular biology methods anneals with chromosome preparations and is detected by fluorescence has gained more attention (Roa and Guerra 2012; Garcia et al. 2014b; Roa and Guerra 2015).

The aim of this study was to assess the current cytogenetic knowledge of Cerrado plants, aiming to provide a consistent database that didn’t exclude any attribute and diagnose recent progress in the field.

## Methods

### Data compilation

Plant names in the Cerrado plant list (Sano et al. 2008) and the cytogenetic literature were checked according to the Brazilian Flora 2020 site (REFLORA 2016) through the www.plantminer.com app (Carvalho et al. 2010). In order to get to the original cytological sources, websites in Table 1 were used. The information in the primary source was organized in the following fields: family (APG 2016), genus, original name as reported in the publication (field = name_on_source), accepted name following the online Brazilian Flora 2020 (field = accepted_name), place of sampling (field = provenance), distribution of C-, Chromomycin A_3_, AgNOR-bands (fields = C-bands, CMA_bands, AgNOR), availability of images (field = images), karyotype formula (field = karyotype), FISH sites position (field = FISH), genome size (field = C-value), meiotic or gametophyte chromosome number (field = meiosis-n), sporophytic chromosome number-2*n* (field = 2*n*), level of ploidy (field = ploidy), average chromosome size (field = ACS), Total chromosome length (field = TCL), Total chromosome area (field = TCA), reference, and authors’ affiliation (field = affiliation). Metadata were gathered partially by scripts from search engines as Scopus (Elsevier) and Crossref (PILA).

**Table 1.** Websites used for the search of literature.

Name	URL
Chromosome Counts Database*	ccdb.tau.ac.il
Plant DNA C-values Database*	data.kew.org/cvalues
International Organization of Plant Biosystematists	www.iopb.org
Scholar Google	scholar.google.com
Scopus	www.scopus.com
Biodiversity Heritage Library	biodiversitylibrary.org
JSTOR	www.jstor.org
Real Jardín Botánico CSIC	bibdigital.rjb.csic.es/spa/index.php
Naturalis Biodiversity Center	www.repository.naturalis.nl
Botanicus Digital Library	www.botanicus.org
Smithsonian Contributions to Botany	repository.si.edu/handle/10088/6943
Crossref	www.crossref.org

*citation in text, data taken from available primary sources.

### Data statistics

All statistics and the online database were performed in R (R Core Team 2016). Packages used were shiny (Chang et al. 2016), shinyjs (Attali 2016), shinydashboard (Chang 2015), ggplot2 (Wickham 2009), plyr (Wickham 2015a), dplyr (Wickham and Francois 2016), stringr (Wickham 2015b), data.table (Dowle et al. 2015), DT (Xie 2015), mongolite (Ooms 2016), gtools (Warnes et al. 2015), robustHD (Alfons 2016) and scales (Wickham 2016). Several frequencies were determined, such as: employed techniques or country of author affiliation per decade; chromosome number (2*n*), genome size, TCL and TCA per taxa. Where not available, TCL and TCA were measured from photos by ImageJ (Schneider et al. 2012). Due to the scarcity of genome size data, a correlation analysis between TCA/TCL and known genome sizes was performed in order to have a rough idea of genome size in pg based on TCL or TCA. In accessions without 2*n* number, they were calculated from meiosis or pollen observations (n).

Even n numbers are more common than odd ones because of the prevalence of even 2n that eventually undergo duplication (Otto and Whitton 2000; Rice et al. 2015). As expected, this trend is more pronounced for high 2n which are usually the result of whole genome duplication events, but may vary by dysploidy. Accessions were subdivided in two groups according to n under or over the mode (n) (low 2n, high 2n); and the even/odd ratio for each group was calculated in order to assess indirectly the degree of polyploidy/dysploidy in each major clade.

## Results

### Coverage

A subset of 38.9% (4,590 taxa) of the Cerrado Plant list was searched, including the 50 richest genera and 273 genera with only one species. Information of 1,431 accessions, from 366 available primary sources, were included in the database. 702 (16.8%) species of our searched sample (4182) had any cytogenetic data and about 500 of them had at least one accession collected in Brazil. Complete lack of information happens for 70% of the single-species genera addressed (Suppl. material 1, 2, 3). The genera *Hyptis* Jacq., 1787 (Lamiaceae), *Stachytarpheta* Vahl, 1804 (Verbenaceae), *Microlicia* D.Don, 1823 (Melastomataceae), *Leiothrix* Ruhland, 1903 (Eriocaulaceae) and *Ditassa* R.Br., 1809 (Apocynaceae) may be considered orphan because they are among the 50 most diverse in Cerrado, but almost no chromosome numbers are known for them (Suppl. material 1). At the family level Eriocaulaceae (Monocots – Commelinids), Lamiaceae, Apocynaceae (Core eudicots-Superasterids-Asterids-Lamiids), Polygalaceae (Core eudicots-Superrosids-Fabids) and Lauraceae (Magnoliids) are also poorly studied (Suppl. material 2), while among the major clades (previously in parentheses), studies for Magnoliids are scarce. Every figure and table is interactive in the database website (http://cyto.shinyapps.io/cerrado).

For the richest and best sampled families, the following modal 2n counts were observed: Oxalidaceae 2n = 12, Lythraceae 2n = 30, Sapindaceae 2n = 24, Solanaceae 2n = 24, Cyperaceae 2n = 10, Poaceae 2n = 20, Asteraceae 2n = 18 and Fabaceae 2n = 26 (Suppl. material 2). At the genus level, *Paspalum* L., 1759 (Poaceae), *Cuphea* P.Browne, 1756 (Lythraceae) and *Rhynchospora* Vahl, 1805 (Cyperaceae) stand among the best studied with ca. 50% of coverage. For ca. 20% of species in most major clades, the chromosome number has been studied (Suppl. material 3).

### Techniques chronology

**Figure 1.**
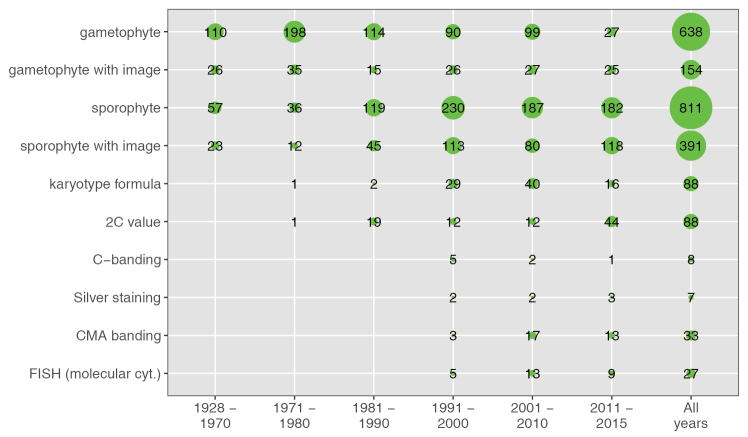
Chronology of cytogenetic techniques or parameters published for Cerrado plant species. Numbers of accessions in the database.

Frequencies of use of karyological techniques show that the first cytogenetic analysis were mainly based on meiosis from 1928 on, and subsequently by 1990 the analysis of mitosis (sporophyte) gained more prevalence. Measurements of karyotype formula (morphology) achieved significance after the 1990’s. A trend to include images in publications, only with a fall during the 2000’s decade was observed. Sophisticated techniques that show a substantial rise from the 2000’s decade are the estimation of the C-value, CMA banding (including C-CMA banding) and FISH. However, they have been applied to a limited number of species, ranging from 0.5 to 2.3% of the 1,431 entries in the database (Fig. 1).

### Author affiliations

**Figure 2.**
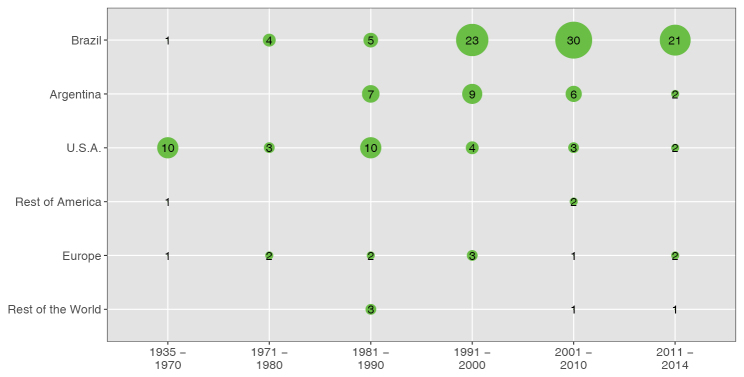
Chronology of author affiliation for Cerrado plant species collected in Brazil. Numbers of references in the database.

In order to track affiliation of authors over time, they were clustered by decade in a time-line. For accessions collected in Brazil [774 (54%)] the country of affiliation of the author showed an increase in the prevalence of Brazilian based research after the 1990’s. Other significant contributions for Brazilian samples have been made by Argentinian based authors (Fig. 2). Most publishing authors for the 1935 – 1970 period were affiliated in the U.S.A, and afterwards, in Brazil. Among the authors with more entries, Coleman, Irwin and Turner studied in the 1935 – 1970 period mainly Asteraceae and Fabaceae. In the 1970 decade, Coleman studied Fabaceae and Nassar, Euphorbiaceae. In the 80’s and 2000’s Graham studied Lythraceae, and in the 90’s Guerra published mainly data on Cyperaceae, Orchidaceae and Velloziaceae. In the current decade, Félix published mainly about Euphorbiaceae and Fabaceae (Suppl. material 4). Accordingly, those authors correspond to the most important nodes in the co-author network (database website).

## Chromosome numbers and genome size

**Figure 3.**
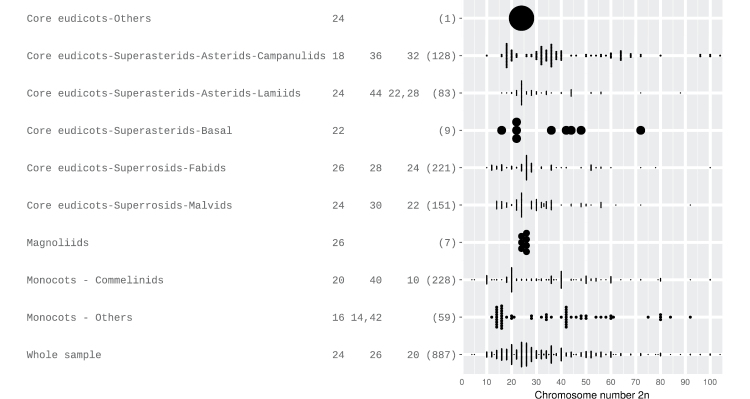
Dot-plot of observed and calculated 2n numbers for Cerrado plant species considering APG major clades. The three most common numbers (frequency ties separated by comma) and sample size in parentheses are indicated. Dot size varies depending on the maximum frequency of each group.

699 (16.1%) species of the searched list have known chromosome numbers with a general median of 2*n* = 28. Fig. 3 shows two ploidy related prevalent chromosome numbers; neopolyploids in Commelinids, which corresponds to *Paspalum* counts (2n = 20 and 40), and several paleopolyploid genera as *Habenaria* with 2n = 42 (Monocots) and *Mikania* with 2n = 36 (Eudicots-Asterids-Campanulids). An unusual conspicuous high frequency of 2n = 36 for the whole sample, corresponds mainly to genera *Manihot* Mill., 1754 (Euphorbiaceae), Tibouchina Aubl., 1775 (Melastomataceae) and *Mikania* Willd., 1803 (Asteraceae). Intraspecific chromosome number variation occurs in 14.3% of species, being polyploidy the cause in 88% of them. At the genus level, most high chromosome numbers are multiples of one of the lowest modal numbers of the genus, i.e. base numbers (database website). The even/odd ratio of n is higher for the groups with “*n* greater than the mode” in almost every clade, as expected, except for Asterids-Campanulids (ratio 1.5), while Asterids-Lamiids shows the greatest bias towards even numbers (ratio 6.5) in karyotypes with n greater than the mode (Table 2).

**Table 2.** Even/odd ratio for haploid (n) numbers considering mode of n.

Clade or group	n even/odd ratio	
	Greater	Lesser
	than n mode	
Monocots - Commelinids	3.47	0.40
Other monocots	4.00	0.12
Core eudicots-Superasterids-Asterids-Lamiids	6.50	1.00
Core eudicots-Superasterids-Asterids-Campanulids	1.50	1.57
Core eudicots-Superrosids-Malvids	3.22	0.50
Core eudicots- Superrosids-Fabids	3.40	0.56

**Figure 4.**
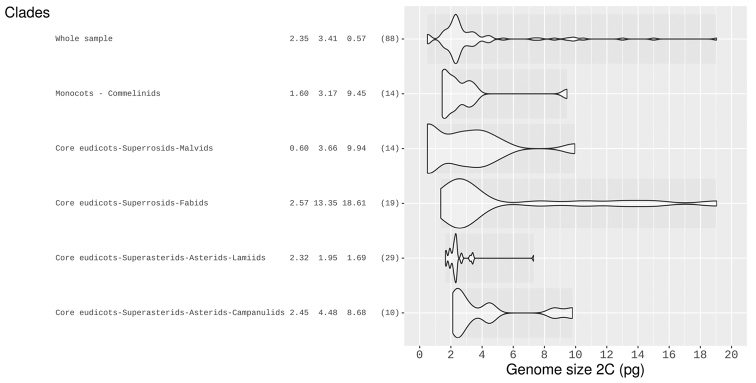
Violin-plots for genome sizes in pg for Cerrado angiosperms. Y-axis contains clades, three highest peaks of the Gaussian kernel density and sample size. Bandwidth of the density estimator customizable in http://cyto.shinyapps.io/cerrado

Only 88 C-values were found for the taxa of Cerrado, most of them ranging from 2C = ca. 1.5 pg to ca. 3.5 pg with a median value of 2.38 pg (Fig. 4). That corresponds to 1.25% of the addressed species list. For a sample of known TCL (or TCA) and C-value, a correlation analysis, although significant, resulted in low r^2^ values (weak to moderate). Applying a linear model to predict 2C-value based on TCL, resulted in prediction intervals of ca. 2pg. TCL and TCA values together with some predicted 2C- values are shown in Suppl. material 5, 6 and the database website.

## Discussion

There is strong interest for genomics and genetics of Cerrado species (Souza et al. 2016). Knowledge of basic data as chromosome number, ploidy level and genome size is critical for the selection of model species (Kelly et al. 2012). Though recent cytogenetic studies tend to incorporate phylogenetic frameworks and correlate cytogenetic with ecological variables, this kind of studies are scarce for Cerrado (Glick and Mayrose 2014; Suda et al. 2015; Silveira et al. 2016). As noted by Guerra (1990), previous to the 1990 decade, cytogenetic studies of authors from U.S.A. prevailed. After that period, mitotic analysis increased compared to meiotic studies, which relate to the establishment of cytogenetic laboratories allowing the treatment with antimitotics of root meristems. Still, the amount of chromosome number data for species occurring in Cerrado is lower than the world total, 16.1 vs. 20% (Rice et al. 2015) and several “orphan” genera were identified, such as *Stachytarpheta* (Verbenaceae), *Microlicia* (Melastomataceae) and *Leiothrix* (Eriocaulaceae). Unlike other techniques, C-banding and silver staining studies did not increase in recent decades, which might be related to low repetitiveness of methods (Guerra 1990). Despite recent efforts, the percentage of known Cerrado C-values is still lower than the 2.1% for all angiosperms (Garcia et al. 2014a). Data of TCA, and TCL for a larger number of species were intended to be used as proxy for genome size after an analysis that resulted in weak to moderate significant correlation with 2C-value in pg. Though they can give a rough estimate of the magnitude of the genome, the prediction interval is high considering most genomes fall within a 2 pg range.

Discontinuities in chromosome numbers seen as multimodal distributions indicate that higher numbers are polyploids generated by genome duplication. A polyploid origin for most high n numbers is also evidenced by the high (> 1) even/odd ratios of *n* (Rice et al. 2015). In contrast, low even/odd value (for *n* greater than the mode) is rare and suggests increased dysploidy and/or low frequency of polyploidy, compared with groups that follow the opposite general trend.

## Conclusion

The Database of Cytogenetics of Cerrado plants and its website is presented, making an important step in facilitating access to most known cytogenetic attributes (http://cyto.shinyapps.io/cerrado/). The amount of chromosome number data for Cerrado is lower than the world total. The chromosome number, is still lacking in several rich genera and families, and therefore, they can be considered “orphan”. This complete lack of information also happens for 70% of the single-species genera addressed. Analysis of chromosome numbers at several taxonomical levels revealed a straightforward relationship between polyploidy and high chromosome numbers. Regarding other techniques, like heterochromatin staining, molecular cytogenetics and C-value estimation, they have been applied to a very small percentage of species, however, those studies have been steadily increasing since 2000. Though cytogenetic data are the basis for some evolutionary and ecological studies, there is a lack of those kind of interdisciplinary studies for Cerrado.

## Supplementary materials

Supplementary material 1 Summary statistics for genera Data type: cytogenetic attributes Explanation note: more details in http://cyto.shinyapps.io/cerrado This dataset is made available under the Open Database License (http://opendatacommons.org/licenses/odbl/1.0/). The Open Database License (ODbL) is a license agreement intended to allow users to freely share, modify, and use this Dataset while maintaining this same freedom for others, provided that the original source and author(s) are credited.Fernando Roa, Mariana Pires de Campos Telles

Supplementary material 2 Summary statistics for families Data type: cytogenetic attributes Explanation note: more details in http://cyto.shinyapps.io/cerrado This dataset is made available under the Open Database License (http://opendatacommons.org/licenses/odbl/1.0/). The Open Database License (ODbL) is a license agreement intended to allow users to freely share, modify, and use this Dataset while maintaining this same freedom for others, provided that the original source and author(s) are credited.Fernando Roa, Mariana Pires de Campos Telles

Supplementary material 3 Summary statistics for major clades Data type: cytogenetic attributes Explanation note: more details in http://cyto.shinyapps.io/cerrado This dataset is made available under the Open Database License (http://opendatacommons.org/licenses/odbl/1.0/). The Open Database License (ODbL) is a license agreement intended to allow users to freely share, modify, and use this Dataset while maintaining this same freedom for others, provided that the original source and author(s) are credited.Fernando Roa, Mariana Pires de Campos Telles

Supplementary material 4 Authors with more publications of cytogenetic data of *Cerrado* plants per decade Data type: affiliation and taxa – categorical data table Explanation note: more details in http://cyto.shinyapps.io/cerrado Copyright notice: This dataset is made available under the Open Database License (http://opendatacommons.org/licenses/odbl/1.0/). The Open Database License (ODbL) is a license agreement intended to allow users to freely share, modify, and use this Dataset while maintaining this same freedom for others, provided that the original source and author(s) are credited.Fernando Roa, Mariana Pires de Campos Telles

Supplementary material 5 Figure S1. Total chromosome length for *Cerrado angiosperms* Data type: Histogram Explanation note: See text for details. This dataset is made available under the Open Database License (http://opendatacommons.org/licenses/odbl/1.0/). The Open Database License (ODbL) is a license agreement intended to allow users to freely share, modify, and use this Dataset while maintaining this same freedom for others, provided that the original source and author(s) are credited.Fernando Roa, Mariana Pires de Campos Telles

Supplementary material 6 Figure S2. Total chromosome area for *Cerrado angiosperms* Data type: Histogram Explanation note: See text for details. This dataset is made available under the Open Database License (http://opendatacommons.org/licenses/odbl/1.0/). The Open Database License (ODbL) is a license agreement intended to allow users to freely share, modify, and use this Dataset while maintaining this same freedom for others, provided that the original source and author(s) are credited.Fernando Roa, Mariana Pires de Campos Telles
