# Supplementary material for: The Cerrado (Brazil) plant cytogenetics database
Source: Comp Cytogenet. 2017 Apr 25;11(2):285–97. doi: 10.3897/CompCytogen.11(2).11395 (PMC5596992; doi:10.3897/CompCytogen.11(2).11395)

Fig. S1. Total chromosome length for Cerrado angiosperms. * Some predicted 2C-values based on a model generated with known 2C and TCL.

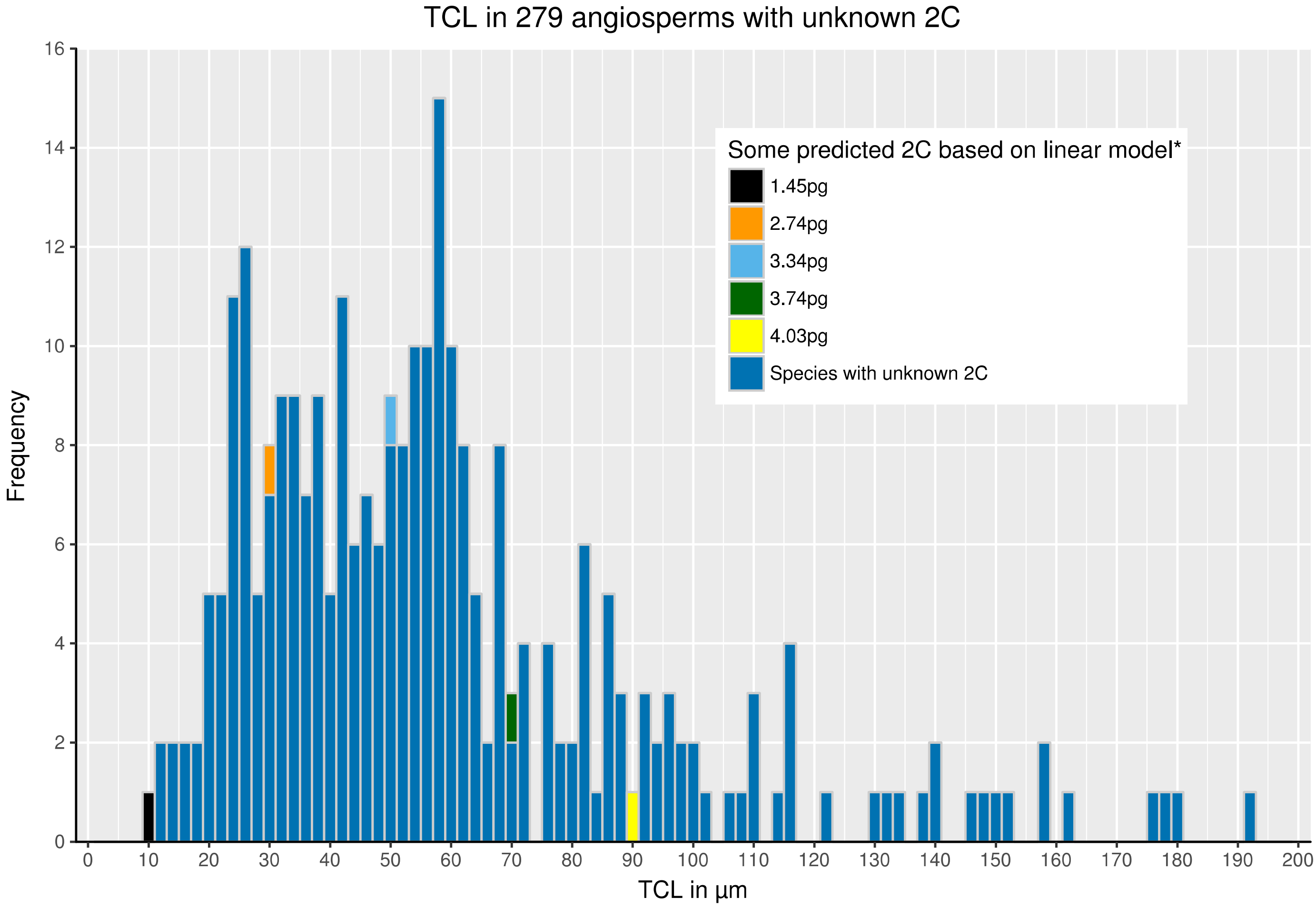

Supplement: Supplementary material 5 — Figure S1. Total chromosome length for Cerrado angiosperms [file comparative_cytogenetics-11-285-s005.pdf]
